# In Search of TGCT Biomarkers: A Comprehensive *In Silico* and Histopathological Analysis

**DOI:** 10.1155/2020/8841880

**Published:** 2020-11-06

**Authors:** Dora Raos, Jure Krasic, Silvija Masic, Irena Abramovic, Marijana Coric, Bozo Kruslin, Ana Katusic Bojanac, Floriana Bulic-Jakus, Davor Jezek, Monika Ulamec, Nino Sincic

**Affiliations:** ^1^Department of Medical Biology, University of Zagreb School of Medicine, Šalata 3, 10000 Zagreb, Croatia; ^2^Scientific Group for Research on Epigenetic Biomarkers, University of Zagreb School of Medicine, Šalata 3, 10000 Zagreb, Croatia; ^3^Scientific Centre of Excellence for Reproductive and Regenerative Medicine, University of Zagreb School of Medicine, Šalata 3, 10000 Zagreb, Croatia; ^4^Ljudevit Jurak Clinical Department of Pathology and Cytology, Sestre Milosrdnice University Hospital Center, Vinogradska Cesta 29, 10000 Zagreb, Croatia; ^5^Department of Pathology and Cytology, University Hospital Centre Zagreb, Kišpatićeva Ulica 12, 10000 Zagreb, Croatia; ^6^Department of Histology and Embryology, University of Zagreb School of Medicine, Šalata 3, 10000 Zagreb, Croatia; ^7^Department of Pathology, University of Zagreb School of Dental Medicine and School of Medicine, Gundulićeva Ulica 5, 10000 Zagreb, Croatia

## Abstract

Testicular germ cell tumors (TGCTs) are ever more affecting the young male population. Germ cell neoplasia *in situ* (GCNIS) is the origin of TGCTs, namely, seminomas (SE) and a heterogeneous group of nonseminomas (NS) comprising embryonal carcinoma, teratoma, yolk sac tumor, and choriocarcinoma. Response to the treatment and prognosis, especially of NS, depend on precise diagnosis with a necessity for discovery of new biomarkers. We aimed to perform comprehensive *in silico* analysis at the DNA, RNA, and protein levels of six prospective (*HOXA9*, *MGMT*, *CFC1*, *PRSS21*, *RASSF1A*, and *MAGEC2*) and six known TGCT biomarkers (*OCT4*, *SOX17*, *SOX2*, *SALL4*, *NANOG*, and *KIT*) and assess its congruence with histopathological analysis in all forms of TGCTs. Cancer Hallmarks Analytics Tool, the Search Tool for the Retrieval of Interacting Genes/Proteins database, and UALCAN, an interactive web resource for analyzing cancer OMICS data, were used. In 108 TGCT and 48 tumor-free testicular samples, the immunoreactivity score (IRS) was calculated. SE showed higher frequency in DNA alteration, while DNA methylation was significantly higher for all prospective biomarkers in NS. In GCNIS, we assessed the clinical positivity of *RASSF1* and *PRSS21* in 52% and 62% of samples, respectively, in contrast to low or nil positivity in healthy seminiferous tubules, TGTCs as a group, SE, NS, or all NS components. Although present in approximately 80% of healthy seminiferous tubules (HT) and GCNIS, *HOXA9* was diagnostically positive in 64% of TGCTs, while it was positive in 82% of NS versus 29% of SE. Results at the DNA, mRNA, and protein levels on putative and already known biomarkers were included in the suggested panels that may prove to be important for better diagnostics of various forms of TGCTs.

## 1. Introduction

Testicular germ cell tumors (TGCTs) represent the most common malignancy among males between 15-45 years of age in Caucasian populations [1]. Although they make up around 1% of all newly diagnosed neoplasms [2], according to GLOBOCAN 2018, TGCT incidence will increase by the year 2040 to a yearly incidence of 85,635 new cases worldwide [3]. Mortality is stable in most high-resource countries, with the curative rate of TGCT being above 95% due to advances in treatment strategies and surgical techniques [4]. Still, some populations like Croatian are experiencing a rise in mortality [5]. Furthermore, they have an exceptional effect on the demographic and socioeconomic status of the affected population [6].

TGCTs are a very heterogeneous group of neoplasms divided into seminomas (SE) and nonseminomas (NS). Pure SE are the most frequent and account for about 55% of all TGCTs in patients with a median age of 35. NS accounts for about 45% of all diagnosed TGCTs in patients with a median age of 25 [7]. A TGCT precursor lesion is GCNIS (germ cell neoplasia *in situ*) [8] which arises in the seminiferous tubule from blocked or arrested primordial germ cells (PGCs). PGCs are pluripotent cells that rise from the epiblast during embryonic development. As a consequence of genetic and (micro) environmental events, this cell population may be blocked or arrested in differentiation and transformed into GCNIS [9].

SE always consists of undifferentiated cells and arises as a pure form [10]. NS consists of four different histological subtypes: embryonal carcinomas (EC), teratoma (TE), yolk sac tumor (YST), and choriocarcinoma (CH). EC consists of embryonal carcinoma cells (ECCs) that are highly similar to embryonic stem cells (ESCs) [11]. ECCs may differentiate along an embryonal line into TE or along an extraembryonal line into YST or CH [12]. NS subtypes rarely appear as pure forms, but rather as mixed germ cell tumors (MGCT) consisting of different components, sometimes including SE as well [13].

Despite histological heterogeneity, most TGCTs have the same genetic hallmark and additional genetic material on chromosome 12p [14]. Its biological function in the tumor is still unknown [15]. GCNIS lacks amplification of the 12p chromosome, which indicates that oncogenes and tumor suppressor genes on 12p are involved in the development of TGCT from GCNIS [14]. Specific gene mutations are also associated with TGCT [16]. For example, KIT mutation is the most common mutation in TGCTs found in about 25% of SE but is rare in NS [17].

Aside from genetic anomalies, epigenetic changes seem to play an important role in the development of TGCTs [18]. Indeed, epigenetic mechanisms, i.e., DNA methylation, chromatin remodeling, and microRNAs (miRNAs), are the focus of contemporary TGCT research [19]. Some miRNAs were reported as tumor suppressors and others as oncomiRs [20]. Considering DNA methylation, several studies have shown that SE exhibits strong global genome hypomethylation, while NS display a hypermethylated genome. Hypermethylation of specific gene promoters in NS could be related to the differentiation level of the tumor tissue. Different methylation patterns of NS and SE may be because they arise from the cells that during normal development switch from an undermethylated genome to a methylated genome [21].

Immunohistochemical analysis is the main molecular tool used in diagnostics of TGCT, especially to differentiate the components of TGCT [22]. As Chovanec and Cheng [23] have reported, novel molecular biomarkers are investigated and novel pathology methods are expected to make progress in TGCT diagnostics in both tissue and liquid biopsies [24]. Namely, elevated levels of classical serum biomarkers beta-human chorionic gonadotropin (bHCG), alpha-fetoprotein (AFP), and lactate dehydrogenase (LDH) are detected in less than 50% of patients. In addition, LDH is of low specificity. Consequently, serum biomarkers used in clinics are of limited clinical utility [25]. The current obstacle is the heterogeneity of molecular profiles of various TGCT subtypes, which often show discordance on different molecular levels, e.g., mRNA expression being out of line with DNA methylation or DNA alteration [26]. Until this overrun, molecular analysis on tumor tissue/biopsies without HE and IHC could result in an aggressive TGCT component going undetected. One also has to keep in mind the presence of GCNIS in the samples with their distinct molecular profiles [9].

Based on the critical review of the literature, 12 TGCT-related genes were chosen for this study, six of which are already used in histopathological diagnostics of TGCTs (*OCT4*, *c-KIT*, *NANOG*, *SOX2*, *SOX17*, and *SALL4*) and six as prominent biomarkers (*MGMT*, *RASSF1*, *HOXA9*, *CFC1*, *PRSS21*, and *MAGEC2*). Most of the already known TGCT biomarkers, *OCT4*, *NANOG*, *SOX2*, *SOX17* [27], and *SALL4* [28], are embryonal markers, expressed in primordial germ cells (PGCs) [29]. They are crucial for stem cell specification, maintenance of the somatic stem cell population, gastrulation, and embryo development [30]. After birth, their expression decreases and finally disappears [9]. C-KIT is a transmembrane receptor that is important for the regulation of cell proliferation, survival, and migration. *KIT* along with *KITLG* is expressed in PGCs, and their interaction plays a crucial role in the migration process [31]. Several investigations of tumor suppressor genes *RASSF1* and *MGMT* were concentrated on epigenetic alterations [32, 33]. Still, protein expression of *RASSF1* was not investigated yet, while *MGMT* expression was reported in TGCT. The methylation patterns of *HOXA9* and *CFC1* were already investigated as potential TGCT markers [32], but, to our knowledge, immunohistochemical studies of these two genes on TGCTs have not been reported.

*HOXA9* is a member of homeobox genes (*HOX* genes) and in the healthy tissue has a crucial role in hematopoiesis. Overexpression of *HOXA9* is associated with poor prognosis of acute myeloid leukemia (AML) [34]. *CFC1* is important during embryonal development. Expression of *CFC1* in adult tissue is associated with various cancers, e.g., pancreatic cancer, colon cancer, and gall bladder cancer [35]. The function of *PRSS21* in tumors is still unknown, but it is hypothesized to be a tumor suppressor [36]. To our knowledge, analysis of *PRSS21* protein expression in TGCT has not yet been done, although its DNA methylation and gene expression status in SE and NS are extensively investigated [37]. *MAGEC2* belongs to the *MAGE* family which codes for cancer-testis antigen. They are located on the X chromosome and involved in normal tissue development as well as in tumorigenesis of various tumors [38, 39]. *MAGEC2* expression is restricted to male germ cells and is not found in adult somatic cells. The protein expression of *MAGEC2* in adult testicular tissue was reported as a novel sensitive marker for SE almost 10 years ago [40], while investigations of its mRNA expression and methylation pattern have not been reported yet.

Precise diagnosis of TGCT is of great importance. Diverse histology and morphology of each individual TGCT component present in the patient which is often in the form of a MGCT require different treatments and management of the TGCT components [8] which makes accurate diagnosis be of great importance. Nowadays, the precise diagnosis of different TGCT subtypes and components is performed on hematoxylin-eosin slides on light microscopy with the help of immunohistochemistry [41]. Although a lot of researchers are focused on finding biomarkers, especially in liquid biopsies, that would allow discriminating different TGCT components on a molecular level with high specificity and sensitivity, still, there are no true biomarkers that could replace classic histology and immunohistochemistry in the diagnostics of TGCT.

Therefore, in this study, we have investigated the molecular profile of selected genes in all relevant tissue types, i.e., healthy testicular seminiferous tubule tissue (HT), GCNIS, TGCT, SE, and NS, as well as NS components. We performed comprehensive *in silico* analysis at the DNA and RNA levels of, above-described, six prospective (*HOXA9*, *MGMT*, *CFC1*, *PRSS21*, *RASSF1A*, and *MAGEC2*) and six recognized TGCT biomarkers (*OCT4*, *SOX17*, *SOX2*, *SALL4*, *NANOG*, and *KIT*). We further assessed the congruence of *in silico* results with histopathological analysis in all forms of TGCTs and healthy tissue. The findings of this study could elucidate on which molecular level and between which tissue types TGCT-related genes are discriminative.

## 2. Materials and Methods

### 2.1. Assessment of Interactions and Cancer Hallmarks

To identify functional interactions between selected genes, their coexpression, gene enrichment and functional analysis, the Search Tool for the Retrieval of Interacting Genes/Proteins database (STRING, version 11.0.), and Cytoscape 3.7.2 were used [42]. The STRING database connects genes based on predicted interactions of their protein product, including direct (physical) and indirect (functional) associations. The interactions are determined based on genomic context, experimental data, coexpression, and previous knowledge. For functional enrichment analysis, results with FDA < 0.05 were considered significant.

Cancer Hallmarks Analytics Tool (CHAT) was used to determine the most evident cancer-related process for each selected gene. Based on the determined cancer-related process, selected genes were assigned to the specific hallmark of cancer according to the hallmarks of cancer (HoC) taxonomy of Baker et al. [43].

### 2.2. Ethical Statement

The study was conducted according to the Declaration of Helsinki. The Ethics Committee of School of Medicine University of Zagreb, Sestre Milosrdnice University Hospital Center, and University Hospital Centre Zagreb approved the collection and manipulation of all tissue samples.

### 2.3. Sample Collection

In total, 108 GCNIS-related TGCT samples and nine tumor-free testicular samples were retrieved from the Tumor Registry at Sestre Milosrdnice University Hospital Center and University Hospital Centre Zagreb from a 20-year period (1999-2018). Tumor samples were routinely processed in the pathohistological laboratory and stored in the form of paraffin blocks, which were further analyzed. Three pathologists performed pathologic examination of routine hematoxylin-eosin–stained sections on each case. Cases were reevaluated, and histological subtypes of TGCT tumors were described (EC, YSC, TE, CH, and SE). The extent of the heterogeneous component was assessed as a percentage of the tumor, and a joint committee resolved all doubts.

Out of 108 TGCT archive samples, 52 were SE and 56 were NS (with 41 EC, 29 TE, 27 YST, 20 SE, and 12 CH components being present in various combinations). Seminiferous tubules with preserved spermatogenesis from TGCT samples (ST TGCT) and tumor-free testes (ST TF) were used as the joined control group (HT) [44], since no difference in protein expression between them was found (Supplementary data 1), making in total 48 tumor-free testicular samples.

### 2.4. Analysis of Gene Expression on the Protein Level

Samples were cut at 4 *μ* and deparaffinized. Antigen retrieval was performed with Tris-EDTA (pH 9) or citrate buffer (pH 6). Subsequently, sections were incubated with 10% BSA for 20 minutes. Then, sections were incubated with 12 primary antibodies (Supplementary data 2) overnight at 4°C. Incubation with 3% H_2_O_2_ was performed to block endogenous peroxidase followed by incubation with a secondary antibody (Dako REAL EnVision, K5007). The signal was visualized with chromogen DAB (Dako REAL, K5007). Slides were counterstained with hematoxylin, imbedded, and analyzed under Olympus Bx53. Appropriate positive and negative controls were used in staining.

Three pathologists performed morphometric analysis of the investigated genes for protein expression. A joint committee resolved all disagreements. Expression of proteins was analyzed in components of seminiferous tubules with apparent spermatogenesis, germ cell neoplasia *in situ*, and tumor tissue. Staining signal (brown in color) was noted as nuclear, cytoplasmic, or membranous in tumor cells (TC), depending on the expected protein location. Staining percentage was scored from 0 to 5: 0 (negative TC), 1 (>1-≤10% positive TC), 2 (>10%-≤25% positive TC), 3 (>25%-≤50% positive TC), 4 (>50%-≤75% positive TC), and 5 (>75% positive TC). Intensity of staining was assessed (none-low-medium-high). Semiquantification of protein expression was expressed by the immunoreactivity score (IRS) which was calculated by multiplying staining percentage (0-5) and intensity of staining (0-3) creating a range of 0-15. Statistical analysis of protein expression was performed by GraphPad Prism software (Mann-Whitney test and Kruskal-Wallis with Dunn's multiple comparison test). Results were considered statistically significant when *p* < 0.05.

To calculate diagnostic positivity, a cut-off value of IRS 4 was used, with IRS scores 0-3 being declared negative and scores 4+ being declared positive (at a minimum more than 10% positive cells with medium intensity staining), and the percentage of positive samples was calculated per component and gene of interest.

### 2.5. Gene Expression on the mRNA Level and DNA Methylation Analysis

For a comparative analysis of gene expression between healthy testis tissue and TGCT, data from the UCSC RNA-seq Compendium was used, where TCGA and GTEx samples are reanalyzed (realigned to the hg38 genome; expressions are called using RSEM and Kallisto methods) by the same RNA-seq pipeline. Because all samples are processed using a uniform bioinformatic pipeline, a batch effect due to different computational processing is eliminated. UCSC XENA software was used [45, 46]. Gene expression between the 12 target genes was analyzed in the testis *vs*. tumor tissue, and 165 samples were present in the GTEx study and 154 in the TCGA study. Welch's *t*-test was used to detect statistically significant differences between the two.

SE *vs*. NS comparative analysis of gene expression was done using UALCAN, a comprehensive, user-friendly, and interactive web resource for analyzing cancer OMICS data [47]. Level 3 RNA-seq data and TGCT patient data were obtained from Genomic Data Commons (GDC) (https://gdc.cancer.gov/). Sample gene expression data was matched to the TGCT histological type of 133 TGCT patients. Promoter DNA methylation data was matched to the TGCT histological type. DNA methylation was represented as *β* values, which range from 0 (nonmethylated) to 1 (fully methylated) and are calculated by the ratio of methylated and unmethylated probe signal intensities. Student's *t*-test with considered unequal variance was used to detect statistically significant differences between groups.

UALCAN was used to compare promoter DNA methylation levels between SE and NS. Promoter DNA methylation data were obtained from the TGCT GDC of 133 TGCT patients. Promoter DNA methylation data was matched to the TGCT histological type. Student's *t*-test with considered unequal variance was used to detect statistically significant differences between groups.

### 2.6. Gene Alteration Analysis

From The Cancer Genome Atlas project, 149 TGCT samples were analyzed for alterations in the 12 target genes as well as alterations in the 12p chromosome using cBioPortal for cancer genomics [48]. Nonsynonymous mutation and DNA copy number data were matched with deidentified clinical data and processed through an internal pipeline.

## 3. Results

### 3.1. Protein Interaction, Gene Coexpression, Functional Enrichment, and Cancer Hallmark Analysis

**Figure 1:**
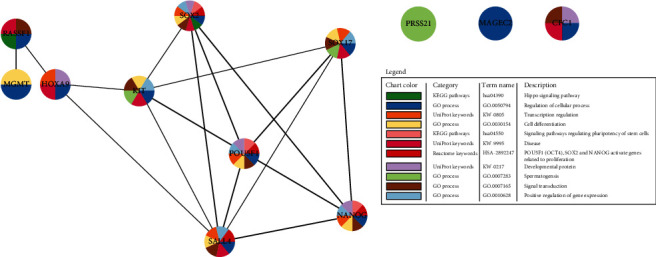
Evidence view of the predicted network of interactions between proteins of interest, by STRING analysis in Cytoscape. Each node represents all proteins produced by a single protein-coding gene locus. Lines of different thickness represent evidence strength used to identify protein-protein interactions. Node colors represent gene enrichment analysis results. Gene coexpression analysis in human tissue has shown ten significant gene coexpressions confirming the interactivity of the investigated genes (Figure 2).

For a better understanding of the biological phenomenon of TGCT, we used STRING to assess the connectivity network of protein expression of genes of interest. The analysis of protein interactions encoded by genes of interest revealed that nine of the 12 investigated proteins comprise an interacting group, with *CFC1*, *PRSS21*, and *MAGEC2* not being connected to it (Figure 1). Functional enrichment analysis has shown significant enrichment in 83 GO terms, eight pathways (KEGG and Reactome), eight UniProt keywords, and ten domains (Supplementary data 3).

**Figure 2:**
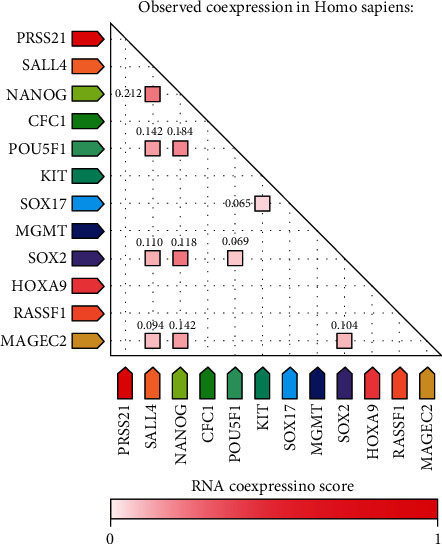
Table of the gene-of-interest coexpression, by STRING v11. Each node represents evidence of intersecting gene coexpression. Lighter and darker hues of red represent weaker and stronger coexpression scores, respectively, based on RNA expression patterns and protein coregulation provided by ProteomeHD.

**Figure 3:**
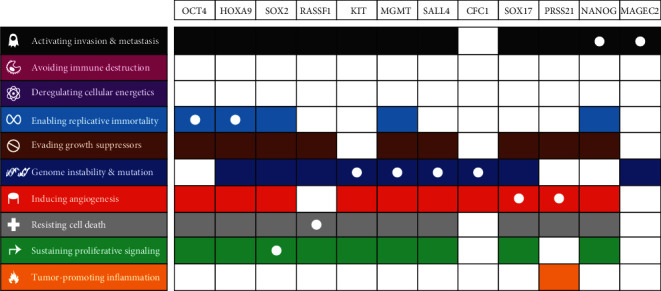
Genes of interest associated with hallmarks of cancer, by CHAT. Each plot represents an analysis of the investigated genes with differently colored sections depicting literature associations with the hallmarks of cancer. White dots represent the strongest association between the hallmarks of cancer and each gene.

For pathways, the descriptions are as follows: hippo signaling pathway, regulating pluripotency of stem cells, and *POU5F1* (encoding for OCT4 protein), *SOX2*, and *NANOG* activating genes related to proliferation; for UniProt keywords: disease, transcription regulation, and developmental protein; and for GO processes: regulation of cellular processes, cell differentiation, spermatogenesis, signal transduction, and positive regulation of gene expression (Figure 2). This shows the involvement of the investigated genes in processes ranging from stemness and differentiation to transcription, signal transduction, and cellular processes highlighting the complexity of TGCT biology. *SOX2*, *OCT4*, *NANOG*, *SALL4*, and *KIT* are involved in the majority of enriched processes (coinciding with the genes with most predicted interactions). Text mining analysis of the selected genes showed their primary involvement in six pathways of cancer hallmarks: genome instability and mutations (*KIT*, *MGMT*, *CFC1*, and *SALL4*), inducing angiogenesis (*PRSS21* and *SOX17*), replicative immortality (*HOXA9* and *OCT4*), invasion and metastasis (*NANOG* and *MAGEC2*), sustaining proliferative signaling (*SOX2*), and resisting cell death (*RASSF1*) (Figure 3).

### 3.2. Testicular Germ Cell Tumor *vs.* Healthy Testicular Tissue

**Figure 4:**
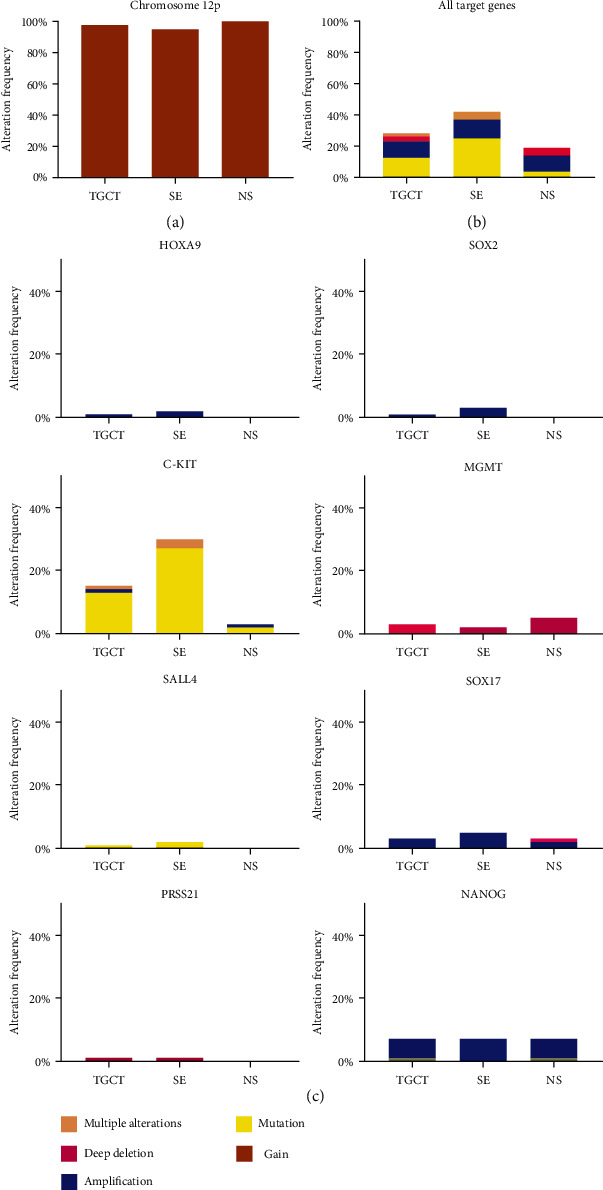
DNA alterations of genes of interest in the testicular germ cell tumor (TGCT), seminoma (SE), and nonseminoma (NS), by cBioPortal. Bioinformatic analysis of DNA alterations across genes of interest in TCGA samples. Alterations are depicted for TGCT as well as for SE and NS, respectively. Total alterations present in analyzed samples, per gene of interest and chromosome 12p, are shown. Genes POU5F1, RASSF1, CFC1, and MAGEC2 are not presented since no alteration was found.

As expected, almost uniform 12p chromosomal gain in 146 of 149 TGCT samples was found. As for the selected genes, alterations have been detected in eight genes (*HOXA9*, *SOX2*, *KIT*, *MGMT*, *SALL4*, *SOX17*, *PRSS21*, and *NANOG*) across 28% of the TGCT samples, with *KIT* alterations alone making up 15% and *NANOG* constituting an additional 7% (Figure 4).

**Figure 5:**
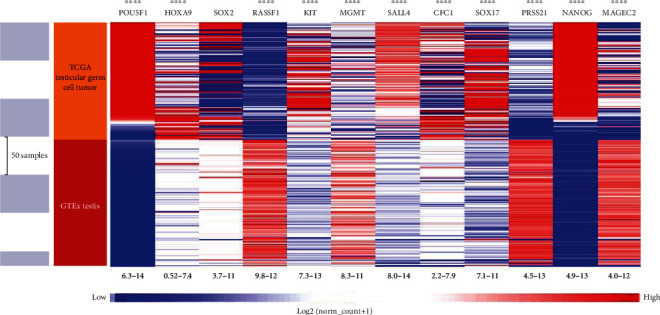
Gene expression on the mRNA level of genes of interest between healthy tissue (HT) and testicular germ cell tumor (TGCT), by XENA. Bioinformatic analysis of gene expression of genes of interest between HT and TGCT samples. The first column shows samples used in the analysis and the second depicts the study of the origin of the samples (TCGA or GTEx) while the third is a genomic heat map of gene expression. Red and blue coloration represents higher and lower mRNA expression, respectively. The difference in gene expression between HT and TGCT is statistically significant in all the investigated genes.

Gene expression at the mRNA level in TGCT *vs*. HT has shown differential expression of all genes of interest, with *RASSF1*, *MGMT*, *PRSS21*, and *MAGEC2* showing higher expression in the healthy tissue than in TGCT, while *POU5F1*, *HOXA9*, *SOX2*, *c-KIT*, *SALL4*, *CFC1*, *SOX17*, and *NANOG* showed higher expression in TGCT (Figure 5).

**Figure 6:**
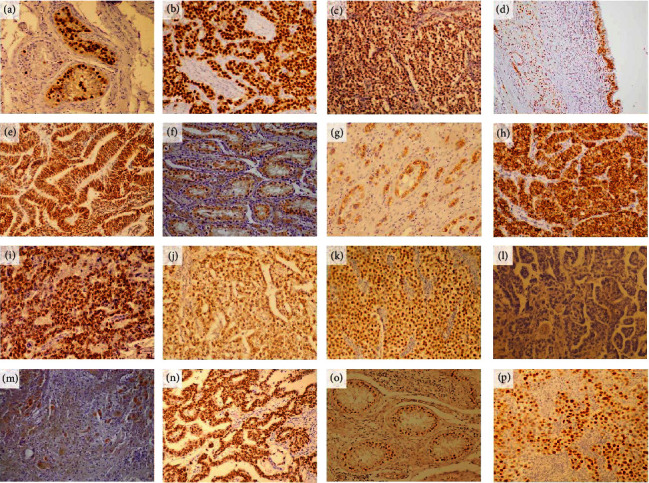
Gene expression on the protein level, by IHC. Expression of genes on a protein level was analyzed by IHC (DAB, hematoxylin, 200x). The level of expression varied from absolute absence to strong IHC signal, depending on the gene analyzed and TGCT component, of which some examples are given in this figure: (a) OCT4 in GCNIS, (b) OCT4 in SE, (c) HOXA9 in SE, (d) HOXA9 in TE, (e) SOX2 in EC, (f) RASSF1A in GCNIS, (g) C-KIT in GCNIS, (h) C-KIT in SE, (i) NANOG in EC, (j) SALL4 in YSC, (k) SOX17 in SE, (l) negative SOX17 in EC, (m) PRSS21 in CH, (n) SALL4 in EC, (o) MAGEC2 in GCNIS, and (p) MAGEC2 in SE.

**Figure 7:**
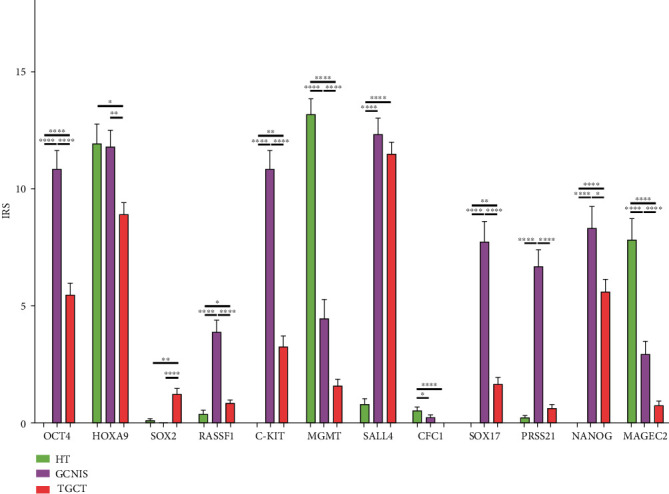
Gene expression on the protein level of genes of interest between healthy tissue (HT), testicular germ cell tumor (TGCT), and germ cell neoplasia *in situ* (GCNIS). Analysis of protein expression of genes of interest between HT, TGCT, and GCNIS, by semiquantitative IHC morphometry, using IRS. Data shown are IRS means ± standarderrors. Statistically relevant differences between these three components are shown with a *p* value. For presenting the *p* value, the asterisk rating system was used: ^∗^*p* < 0.05, ^∗∗^*p* < 0.01, ^∗∗∗^*p* < 0.0002, and ^∗∗∗∗^*p* < 0.0001. Differential gene expression was found across most of the genes of interest.

In clinical TGCT samples, we assessed differential protein expression for most of the investigated genes in HT, GCNIS, TGCT, and TGCT components (Figure 6). Highly expressed *MGMT*, somewhat lowly expressed MAGEC2, and lowly expressed CFC1 have shown the highest IRS in HT, which were significantly lower in GCNIS and even more significantly lower in TGCT. IRS for *OCT4*, *RASSF1*, *c-KIT*, *SALL4*, *SOX17*, *PRSS21*, and *NANOG* were highest in GCNIS and significantly higher in HT and TGCT. The IRS for *SOX2* was highest in TGCT, which was significantly higher in HT and nil in GCNIS. *HOXA9* expression was high and had the lowest IRS in TGCT, which was significantly lower in comparison to its expression in HT and GCNIS (Figure 7).

Histopathological clinical diagnostic analysis (Table 1) has shown, for the first time, that for GCNIS, clinical positivity of RASSF1 and PRSS21 was high, respectively, in 52% and 62% of samples in contrast to that of HT (5% and 2%, respectively). Present in approximately 80% of HT and GCNIS, HOXA9 was diagnostically positive in 64% of TGCTs. Regarding other biomarkers, 91% of HT samples were positive on MGMT, which were much lower than those of GCNIS (30%) and TGCT (11%). For HT, 58% of samples were positive on MAGEC2, which were higher than those of GCNIS (22%) and TGCT (4%). In the GCNIS component, OCT4 has shown clinical positivity in 75% of samples that were higher than those of TGCT (41%), c-KIT in 73% that was higher than that of TGCT (25%), PRSS21 in 62% that was higher than that of TGCT (5%), SOX17 in 55% that was higher than that of TGCT (15%), and NANOG in 60% that was higher than that of TGCT (42%). In the GCNIS component, SALL4 was diagnostically positive in 82% of samples that were somewhat lower than those of TGCT (76%). In TGCT, 12% of samples were positive on SOX2, and nil was positive in HT and GCNIS (Table 1).

In eight of the 12 genes of interest, mRNA level and protein expression correlated in HT and TGCT samples, while *HOXA9*, *RASSF1*, *CFC1*, and *PRSS21* showed inverse protein expression to their mRNA levels.

### 3.3. Seminoma *vs.* Nonseminoma

TGCTs consist of SE and NS that have different clinical presentation; we analyzed and compared DNA alteration frequency, mRNA expression, and DNA methylation separately for SE and NS. Using UALCAN, we were able to compare data on types of DNA alteration (multiple alterations, deep deletion, amplification, mutation, and 12p gain) and assess the percentage of various DNA alteration types for genes of interest. Then, we compared protein expression and its diagnostic positivity for genes of interest, not only in the SE and NS groups but also in all possible components of NS that have different prognostic values.

Analysis showed that 12p gain was present in 95% of SE and 100% of NS samples, making the alteration universal. Selected gene alteration frequency shows 42% of alterations being present in SE and only 18% in NS samples. Among them, *KIT* makes up 30% of alterations in SE and 4% in NS samples and *NANOG* 7% in SE and 7% in NS samples. DNA alterations for other genes were scarce. Among them, *MGMT* showed deep deletions, while *SOX2*, *SOX17*, *NANOG*, and *HOXA9* showed amplifications. There were no data on DNA alterations for *SALL4* and *PRSS21* (Figure 4).

**Figure 8:**
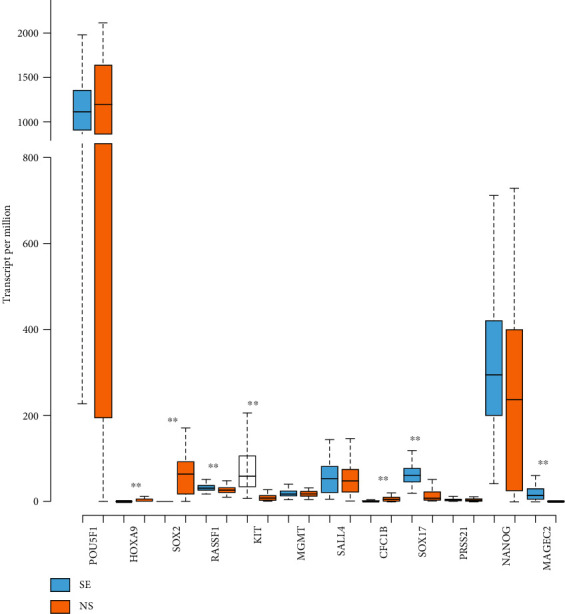
Gene expression on the mRNA level of genes of interest in seminoma (SE) and nonseminoma (NS), by UALCAN. Bioinformatic analysis of gene expression of genes of interest between SE and NS TCGA samples, on the mRNA level. Data shown are transcripts per million, represented as a box and whisker plot including interquartile range with the minimum, 25th percentile, median, 75th percentile, and maximum values. Outliers are excluded from the plot. For presenting the *p* value, the asterisk rating system was used: ^∗^*p* < 0.05, ^∗∗^*p* < 0.01, ^∗∗∗^*p* < 0.0002, and ^∗∗∗∗^*p* < 0.0001. n.s.: no significance. Differential gene expression between SE and NS is shown in multiple genes.

Gene expression has shown no difference in expression between SE and NS in five of the 12 selected genes (*POU5F1*, *MGMT*, *SALL4*, *PRSS21*, and *NANOG*). SE has shown higher expression than NS in *RASSF1*, *KIT*, *SOX17*, and *MAGEC2*, while NS has shown higher expression than SE in *HOXA9*, *SOX2*, and *CFC1B* (Figure 8). Gene coexpression analysis has detected statistically significant coexpression of *SOX17*-*c-KIT* (*p* < 0.001) and *POU5F1*-*SOX2* (*p* < 0.002), which confirms the distinct SE and EC profiles, with SE having *SOX17*-*c-KIT* and EC having *POU5F1*-*SOX2* coexpression.

**Figure 9:**
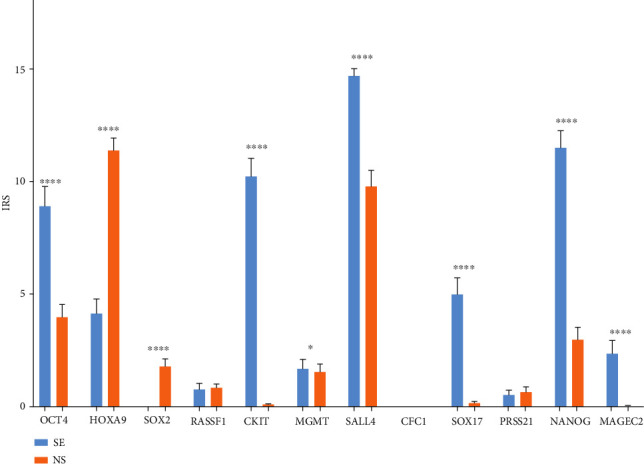
Gene expression on the protein level of genes of interest between seminoma (SE) and nonseminoma (NS). Analysis of protein expression of genes of interest between SE and NS, by semiquantitative IHC morphometry, using IRS. Data shown are IRS means ± standarderrors. Statistically relevant differences between these three components are shown with a *p* value. For *p* values, the asterisk rating system was used: ^∗^*p* < 0.05, ^∗∗^*p* < 0.01, ^∗∗∗^*p* < 0.0002, and ^∗∗∗∗^*p* < 0.0001. Differential gene expression was found across most of the genes of interest.

No difference was found in IRS of RASSF1, CFC1, and PRSS21 between SE and NS components. SE had significantly higher IRS for OCT4, KIT, MGMT, SALL4, SOX17, NANOG, and MAGEC2 when compared to NS, while NS components had higher IRS for HOXA9 and SOX2 (Figure 9). Histopathological assessment of diagnostic positivity in clinical samples (Table 1) showed that diagnostic positivity of two genes (*HOXA9* and *SOX2*) was found more in samples of NS than in those of SE: 82% for HOXA9 in NS versus 29% in SE and 17% for SOX2 in NS versus 0% in SE. On the contrary, diagnostic positivity for another six genes was found more in SE samples than in NS samples: 68% for OCT4 in SE versus 29% in NS, 98% for SALL4 in SE versus 65% in NS, 79% for KIT in SE versus 0% in NS, 46% for SOX17 in SE versus 1% in NS, 85% for NANOG in SE versus 23% in NS, and 15% for MAGEC2 in SE versus 0% in NS (Figure 9, Table 1).

**Figure 10:**
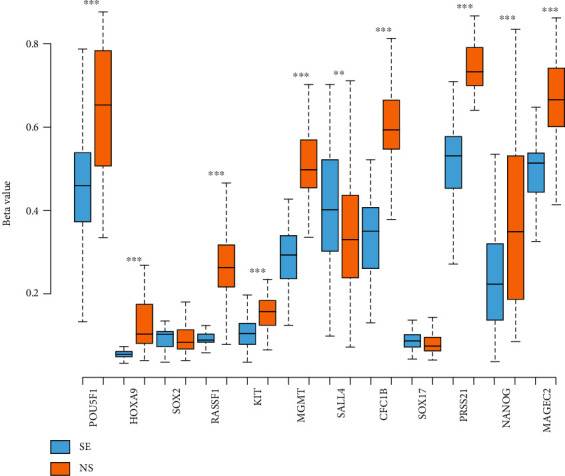
DNA methylation of genes of interest between seminoma (SE) and nonseminoma (NS), by UALCAN. Bioinformatic analysis of DNA methylation of genes of interest in SE and NS TCGA samples using UALCAN. DNA methylation is shown as *β* values, represented as a box and whisker plot including interquartile range with the minimum, 25th percentile, median, 75th percentile, and maximum values. For presenting the *p* value, the asterisk rating system was used: ^∗^*p* < 0.05, ^∗∗^*p* < 0.01, ^∗∗∗^*p* < 0.0002, and ^∗∗∗∗^*p* < 0.0001. n.s.: no significance. Differential DNA methylation is shown across multiple genes.

No difference in DNA methylation was observed in only *SOX2* and *SOX17* between SE and NS samples. The other selected genes show general hypomethylation in SE and hypermethylation in NS samples, apart from *SALL4* which presented the opposite pattern (Figure 10).

**Figure 11:**
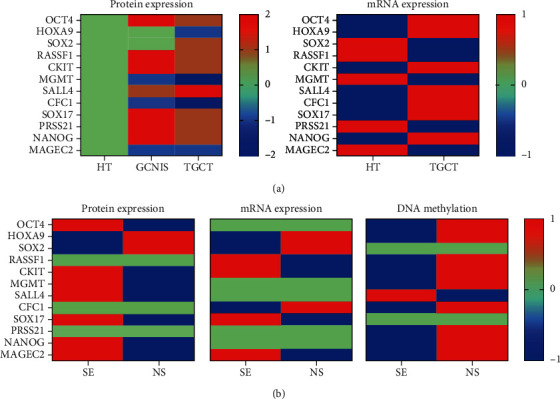
The heat map indicates differences in gene expression between compounds. (a) Protein and mRNA expression of the selected genes in healthy tissue (HT), germ cell neoplasia in situ (GCNIS), and testicular germ cell tumor (TGCT). The protein expression in HT is presented as normal expression (0), while protein expression in GCNIS and TGCT is characterized as increased (2) or decreased (-2) based on the comparison with the expression in HT. The heat map indicates differences in mRNA expression between HT and TGCT, where decreased mRNA expression is characterized as -1 and increased as 1. (b) Protein expression, mRNA expression, and DNA methylation of the selected genes in seminoma (SE) and nonseminoma (NS). No difference in the gene expression between NS and SE is presented as 0, while increased gene expression is presented as 1 and decreased gene expression is presented as -1 in SE and NS based on the comparison between these two entities.

Coherence in DNA methylation levels, mRNA expression, and protein expression was found only for *c-KIT* and *MAGEC2* with lower DNA methylation and higher expression in SE and a higher DNA methylation level and lower expression in NS (Figure 11). For *HOXA9*, lower expression (RNA and protein) in SE than in NS was out of line with DNA methylation being also lower in SE and higher in NS. Considering *SOX2*, expression was lower in SE than in NS, but DNA methylation levels were similar, while for *SOX17*, expression was higher in SE and DNA methylation levels were also similar. For *PRSS21*, no differences in expression were found, although DNA methylation level was higher in NS. Considering *OCT4*, *MGMT*, and *NANOG*, the mRNA level was similar in SE and NS, although protein expression and DNA methylation were higher and lower, respectively, in SE and *vice versa* in NS. Protein expression and DNA methylation levels of *SALL4* in SE were higher than those in NS, but mRNA levels were similar in SE and NS. *CFC1* DNA methylation level and mRNA expression in SE were lower than those in NS, but protein expression was similar.

### 3.4. Individual Nonseminoma Components

**Figure 12:**
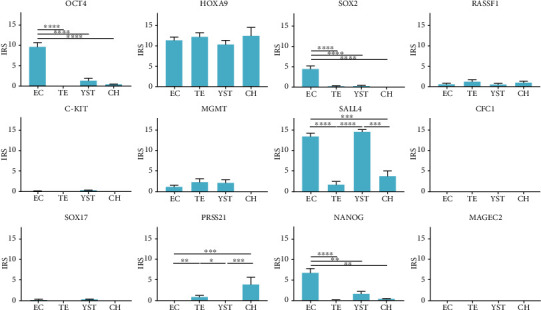
Gene expression on the protein level of genes of interest between nonseminoma (NS) components. Analysis of protein expression of genes of interest between NS components, by semiquantitative IHC morphometry, using the immunoreactivity score (IRS). Data shown are IRS means ± standarderrors. Statistically relevant differences between these three components are shown with a *p* value. The asterisk rating system was used: ^∗^*p* < 0.05, ^∗∗^*p* < 0.01, ^∗∗∗^*p* < 0.0002, and ^∗∗∗∗^*p* < 0.0001. Differential gene expression was found across most of the genes of interest.

The protein expression of NS components is as heterogeneous as the components themselves. The EC component has shown increased protein expression of *OCT4*, *SOX2*, and *NANOG*. EC and YST both have had increased protein expression of *SALL4*, while all four components have had a high level of protein expression in *HOXA9*. CH has shown increased protein expression in *PRSS21* (Figure 12). Diagnostic positivity of 73% in OCT4, 43% in SOX2, and 51% in NANOG has been found in the EC component. All four NS components have shown diagnostic positivity higher than 70% in HOXA9, while EC and YSC have shown positivity of around 90% in SALL4 while TE and CH have exhibited 10-20% (Table 1).

### 3.5. Proposed Diagnostic Flowchart

**Figure 13:**
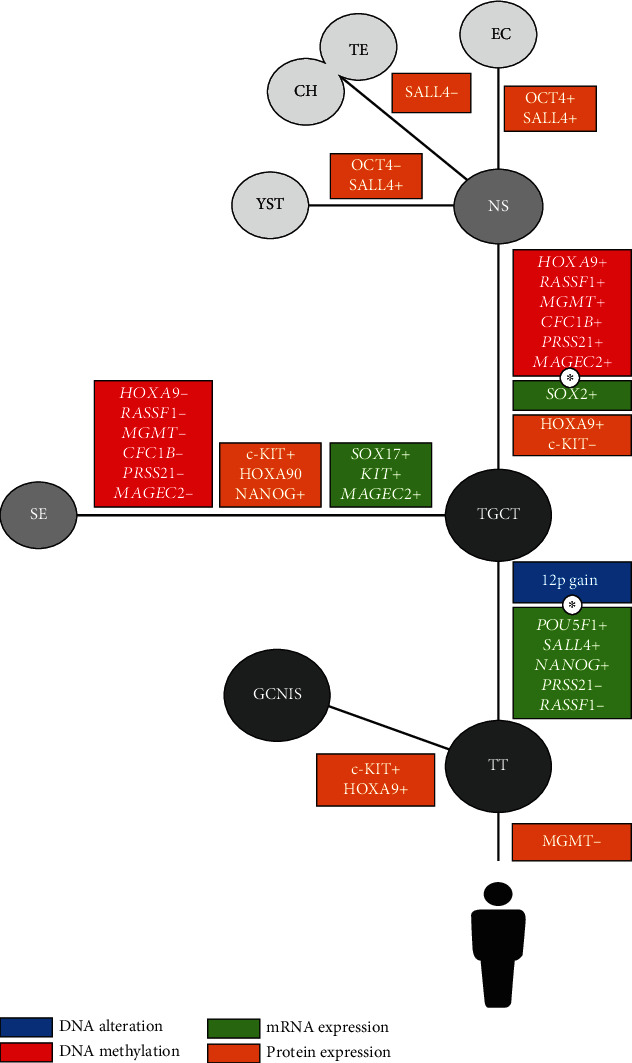
Suggested diagnostic panel for the detection of the testicular tumor (TT) and testicular germ cell tumor (TGCT) and the diagnosis of seminoma (SE), nonseminoma (NS), germ cell neoplasia in situ (GCNIS), and nonseminoma (NS) components. Legend is color-coded for the type of analysis done. The asterisk denotes possible issues with a diagnosis between methods placed because of no information being available for individual NS components, which are known to be very heterogeneous. A complete diagnosis of NS components is not possible without analysis of gene expression on the protein level.

Finally, we incorporated in TGCT diagnostic panels results for 12 genes of interest obtained *in silico* on existing data about the DNA alteration, DNA methylation, and mRNA expression and by histopathological assessment of IRS and percentage of diagnostic positivity in clinical samples. Old and putative biomarkers were assigned to the TGCT group as a whole or a specific TGCT component (GCNIS, SE, NS, EC, TE, YST, and CH) (Figure 13).

From the analyzed DNA alterations, the gain on the 12p chromosome was incorporated in the TGCT panel due to its confirmed overwhelming presence in TGCT samples (Figure 4).

DNA methylation markers were chosen because they expressed the sharp distinction between methylation in NS and demethylation in SE (*HOXA9*, *RASSF1*, *MGMT*, *CFC1B*, *PRSS21*, and *MAGEC2*) (Figure 10).

Among mRNA biomarkers, those that followed the criterion of minimal or no overlap with DNA methylation and no overlap between compared components (HT *vs*. TGCT and SE *vs*. NS) were selected. Therefore, mRNA biomarkers that may be classified for the TGCT group are *OCT4* (*POUF1*), *SALL4*, *NANOG*; for SE, *SOX17*, *c-KIT*, and *MAGEC2*; and for NS, *SOX2* (Figures 5, 8, and 13).

Protein biomarkers were selected if they had at least 70% diagnostic positivity or a great difference in diagnostic positivity between the various TGCT components. For the putative biomarkers, RASSF1 and PRSS21 had high diagnostic positivity in GCNIS versus low diagnostic positivity in all other tissues, including HT. HOXA9 had similar high diagnostic positivity in TGCT as a group and in different NS components, but its IRS and diagnostic positivity were much lower in SE than in NS (Figure 13, Table 1).

## 4. Discussion

Association of *in silico* research on available high-throughput data and histopathological research on clinical samples carried out in our study on 12 genes provided additional data about the old and some putative TGCT biomarkers at the three molecular levels, i.e., the DNA level (DNA structural changes and epigenetic promoter DNA methylation signatures), mRNA level, and protein level. Taking all into account, we organized our results in four panels. The panel specific for the GCNIS that has a highly treatable proposed origin of TGCTs might prove to be of utility for much needed early diagnostics [49]. The next three panels that are discriminating between components of different prognostic and treatment approaches (SE, NS, and components of NS) may be important for early pretreatment diagnostics and the follow-up of treated patients bearing in mind the necessity of a personalized approach and precision medicine [50] (Figure 13).

Out of all the selected genes, *MGMT* was the only one extensively expressed in HT showing a linear, decreasing trend of protein expression from HT to GCNIS and TGCT. Significant higher expression in HT implies that decreasing the activity of this tumor suppressor gene could be crucial for malignant transformation.

### 4.1. Germ Cell Neoplasia *In Situ* Panel

So far, early clinical GCNIS diagnostics are done only *in situ* by classical histology and immunohistochemistry on testicular biopsies in patients with a risk for TGCT development (testis atrophy, infertility, cryptorchidism, or suspicious ultrasound), and no protein marker is specific enough for the detection of GCNIS cells by noninvasive methods such as immunocytochemistry in body fluids [9, 48]. Our results show for the first time a significant increase in two additional markers in GCNIS, i.e., *RASSF1A* and *PRSS21* (testisin) protein expression, in comparison to both healthy seminiferous tubules and TGCT as a group; however, they have not made the 70% diagnostic positivity cut-off for the inclusion in the diagnostic panel. We also confirmed similar differences in protein expression for the already known GCNIS biomarkers: *OCT4*, *SOX17*, *c-KIT*, and *NANOG*. The biomarker *SALL4*, although highly expressed in GCNIS, showed no significant difference in expression in TGCT as a group and therefore was not included in our panel as highly discriminative for GCNIS in comparison to HT and the TGCT group. The combination of expressed genes *HOXA9* and *c-KIT* on the protein level was selected for the GCNIS panel due to their high diagnostic positivity. Indeed, the combination of *HOXA9* and *c-KIT* easily discriminates GCNIS from TGCTs.

### 4.2. Testicular Germ Cell Tumor Panel

Our *in silico* research confirmed an almost ubiquitous gain of the short arm of chromosome 12 in TGCTs that prompted us to include it in the TGCT panel. It is noteworthy that such gain was described only in GCNIS proximal to TGCTs and was absent from distant GCNIS [9]. Among the genes of our interest, *NANOG* is located on the 12p chromosome but its alteration frequency in TGCT was rather low or not detected. We confirmed the highest alteration frequency for *c-KIT*, located on chromosome 4p, while other genes of our interest had a noninvasive rather low frequency of alterations or no alterations. Therefore, DNA alterations of those genes do not seem to be the drivers of TGCTs but are probably only passenger mutations that are estimated to occur in 99.9% of mutations found in cancer [51].

On the mRNA expression level in the TGCTs, *OCT4*, *NANOG*, and *SALL4* displayed great discriminative potential in comparison to those in HT, which is in accordance with their involvement in malignant transformation of the cells [51]. *OCT4* and *NANOG* (stemness or pluripotency genes) in cancer hallmarks mainly enable the tumor replicative immortality and invasion that are crucial for tumor survival and growth [52]. *SALL4* is known for its essential role in maintaining the pluripotent state and self-renewal properties of embryonic stem cells (ESCs) [53]. Unexpectedly, we found out that the reexpression of *SALL4* in an adult testis is primarily associated with genome instability and mutations during tumor progression (invasion and metastasis) which may be important to explain DNA alterations found in TGCTs (Figure 3). Decreased mRNA expression of *RASSF1A* and *PRSS21* confirms the inactivation of tumor suppressor genes [54] in TGCT. Finally, because the differences in mRNA expression of all the mentioned genes between healthy testicular tissue and TGCT were significant, we included them in the TGCT panel.

### 4.3. Seminoma *vs.* Nonseminoma Panel

*In silico*-obtained results on DNA methylation of genes of our interest showed that the promoter of almost every gene was hypermethylated in NS and hypomethylated in SE, which is in congruence with the previously reported results on hypermethylation of the NS genome in comparison to the hypomethylation of the SE genome [55]. Therefore, DNA methylation patterns of *PRSS21*, *CFC1*, *MAGEC2*, *MGMT*, *HOXA9*, and *RASSF1*, which discriminated SE from NS, were included in our panels. Importantly, DNA methylation of *HOXA9*, *CFC1*, *RASSF1*, and *MGMT* has taken together displayed high accuracy in distinguishing SE from NS [32]. *MAGEC2*, a previously reported sensitive protein SE biomarker [40] that has so far not been investigated as a discriminative marker on the DNA methylation level, was now included in our panels at the DNA methylation level.

The only exception among the genes of our interest was the *SALL4* promoter, which was hypomethylated in NS and hypermethylated in SE, but at the mRNA level, no difference was found, implying other types of regulation of *SALL4* gene expression.

We may conclude that the difference in the DNA methylation pattern of a significant number of genes that we described should be discriminative enough for diagnostics of SE versus NS similar to that recently proposed for lung adenocarcinoma where the methylation in *HOXA9* was upregulated in contrast to its downregulation in other three genes [56]. Importantly, microRNAs of the miR-371-373 cluster that were found in patients with germ cell tumors (GTCs) and even in experimental teratomas derived from human genetically modified stem cells in the teratoma assay that should be devoted not only to the confirmation of pluripotency but also to the possible malignancy of such cells [57, 58] have lately been intensively investigated for liquid biopsies and early diagnostics of TGCT, but they do not discriminate between SE and NS as did our results with promoter methylation. Moreover, some technical requirements must be met such as avoidance of hemolysis that may influence microRNA quantitative measurement [59].

Expression of *SOX2*, *KIT*, *SOX17*, and *MAGEC2* genes on the mRNA level also differs significantly between SE and NS. Increased mRNA expression of *SOX2* in NS is in accordance with the theory that NS arises from blocked PGC by retaining an early embryonic marker profile among highly differentiated NS components [10]. Our results of increased mRNA expression of *c-KIT* and *SOX17* in SE make them promising biomarkers on the mRNA level, especially because they are used in routine diagnostics of TGCT by immunohistochemistry [60]. This suggests that the most conventional protein biomarkers should be investigated on all molecular levels for potential use in liquid biopsy diagnostics.

At both protein and RNA levels, we found out that *HOXA9* is significantly more expressed in NS in comparison with SE with clinical positivity in 82% of NS samples in contrast to 29% in SE. However, *HOXA9* expression was not correlated with an assessed highly discriminative potential on the DNA methylation level, suggesting that there is more to the regulation of gene expression than just DNA methylation at work [61]. This discrepancy should be further investigated in order to understand the full role of this gene in SE *vs*. NS development, having in mind that it is primarily included in replicative immortality cancer hallmarks. We confirmed that protein expression of *KIT* is discriminative enough to be used even individually for SE diagnosis, while *SOX17* and *SOX2* could be used in differentiating SE from NS alongside *OCT4* as previously proposed [62]. *MAGEC2*, which was described as a biomarker for SE [40], in our investigation had a significantly higher IRS than that in NS, but the IRS was lower than our cut-off and that suggests further investigation.

### 4.4. Nonseminoma Component Panel

For each single NS component panel, we used only our IHC data because of the lack of data on a single NS component that could be analyzed *in silico* and merged with our data of gene expression on a protein level. In fact, to our knowledge, no database presents data on an individual NS component, but rather for the whole NS group as a single entity, regardless of different biological and clinical features of EC, TE, YST, and CH. This standard of data representation obscures the differences within the NS group that could be further exploited in developing discriminatory biomarkers between them.

Our results confirm *OCT4* and *NANOG* as biomarkers for EC [60] because their expression in other NS components (with the exception of SE components) was not detected or was much lower. Significantly increased expression of *SOX2* was noticed in EC as well, although it was below our IRS cut-off level. However, we may confirm its use in combination with *OCT4* and *NANOG*, already in the clinical practice [62]. According to the literature, clinical practice, and our results, immunohistochemical detection of *SALL4* protein is a good marker for YST in conjunction with the lack of *OCT4*, *NANOG*, and *SOX2* protein expression [63].

Although teratoma is composed of differentiated cells or tissues, it has malignant potential as a part of the NS group and is very rare in its pure form. In addition, in our protein expression panel, there is no unique marker characteristic for this component, which is expected due to the heterogeneity of teratoma components. It seems that microRNAs of the miR-371-373 cluster do not discriminate teratomas and a professional histopathological analysis is still required for the assessment of mature (MT) and immature teratomas (IT) as a pure TGCT form or as an NS component with other histological germ cell subtypes (EC, CH, YST, and S) or with a somatic malignant component (TMSC: carcinoma, pancreatic neuroendocrine tumor, neuroblastoma, rhabdomyosarcoma, rhabdomyosarcoma and liposarcoma, chondrosarcoma, neurogenic sarcoma, chondrosarcoma and neuroectodermal sarcoma, and malignant peripheral nerve sheath tumor) as exemplified by the research of Terenziani et al. [64].

CH is the rarest and most aggressive form of the NS group, whether it occurs in pure or MGCT form. It gives hematogenous metastases very early in tumor progression with a testicular mass being small or even “burned-out.” In our panel, the CH component showed positive expression of *HOXA9* while all the other markers were negative [65].

## 5. Conclusions

Using a combination of standard clinical biomarkers and promising new TGCT biomarkers, we propose a diagnostic flowchart that distinguishes healthy tissue, GCNIS, TGCT, and TGCT individual components from each other, at least at one molecular level of the central dogma axis, and our panels, therefore, represent a proposal for “multianalyte tests that could be used to detect early cancers in a highly sensitive manner” [66]. Among these especially important markers, it seems to be that methylation markers can be relatively easily assessed in all types of biopsies. Our data, which were obtained by bioinformatics for the DNA methylation and mRNA level combined with histopathological protein assessment in clinical samples, have to be further corroborated by corresponding analysis done at all these levels in a single patient. Our results are also important for further investigation of molecular pathogenesis that is especially obscure in the cases where gene expression is in discordance with, e.g., the level of promoter DNA methylation. Therefore, the regulation of gene expression may be influenced by DNA methylation at some other site within the gene that may have a positive correlation between exon methylation and expression levels [67], at some other epigenetic level (RNA interference, histone signatures) or by the direct interaction of a plethora of regulatory proteins with enhancers or silencers. Our functional enrichment analysis revealing nine genes forming a biological group and *MAGEC2*, *PRSS21*, and *CFC1* being unconnected to it implies that there are more related genes in the network, some of which could be potential biomarkers.

## Figures and Tables

**Table 1:** Diagnostic positivity for the selected genes. Depiction of percentage of samples declared diagnostically positive or negative for the investigated components (HT, TGCT, GCNIS, SE, NS, EC, TE, YST, and CH) per gene of interest. Samples with IRS 0-3 were considered diagnostically positive.

Gene	Samples	HT	GCNIS	TGCT	SE	NS	EC	TE	YSC	CH
OCT4	Total	48	59	156	47	109	41	29	27	12
	Positive	0 (0%)	44 (75%)	64 (41%)	32 (68%)	32 (29%)	30 (73%)	0 (0%)	0 (0%)	0 (0%)
HOXA9	Total	41	60	140	48	92	36	25	24	7
	Positive	35 (85%)	48 (80%)	89 (64%)	14 (29%)	75 (82%)	30 (83%)	22 (88%)	17 (71%)	6 (86%)
SOX2	Total	35	58	154	48	106	40	29	26	11
	Positive	0 (0%)	0 (0%)	18 (12%)	0 (0%)	18 (17%)	17 (43%)	0 (0%)	1 (4%)	0 (0%)
RASSF1	Total	41	64	158	49	109	41	29	27	12
	Positive	2 (5%)	33 (52%)	9 (6%)	3 (6%)	6 (6%)	1 (2%)	3 (10%)	1 (4%)	1 (8%)
C-KIT	Total	45	59	154	48	106	40	29	25	12
	Positive	0 (0%)	43 (73%)	38 (25%)	38 (79%)	0 (0%)	0 (0%)	0 (0%)	0 (0%)	0 (0%)
MGMT	Total	45	53	150	48	102	39	27	26	10
	Positive	41 (91%)	16 (30%)	17 (11%)	7 (15%)	10 (10%)	3 (8%)	4 (15%)	3 (12%)	0 (0%)
SALL4	Total	35	60	143	49	94	36	25	25	8
	Positive	1 (3%)	49 (82%)	109 (76%)	48 (98%)	61 (65%)	32 (89%)	3 (12%)	24 (96%)	2 (25%)
CFC1	Total	37	62	151	48	103	40	27	25	11
	Positive	1 (3%)	2 (3%)	0 (0%)	0 (0%)	0 (0%)	0 (0%)	0 (0%)	0 (0%)	0 (0%)
SOX17	Total	43	60	156	48	108	41	29	26	12
	Positive	0 (0%)	33 (55%)	23 (15%)	22 (46%)	1 (1%)	1 (2%)	0 (0%)	0 (0%)	0 (0%)
PRSS21	Total	47	71	156	47	109	41	29	27	12
	Positive	1 (2%)	44 (62%)	8 (5%)	2 (4%)	6 (6%)	0 (0%)	2	0 (0%)	4 (33%)
NANOG	Total	41	57	157	48	109	41	29	27	12
	Positive	0 (0%)	34 (60%)	66 (42%)	41 (85%)	25 (23%)	21 (51%)	0 (0%)	4 (15%)	0 (0%)
MAGEC2	Total	36	65	156	48	108	41	29	26	12
	Positive	21 (58%)	14 (22%)	7 (4%)	7 (15%)	0 (0%)	0 (0%)	0 (0%)	0 (0%)	(0%)

## Data Availability

The raw data supporting the conclusions of this manuscript will be made available by the corresponding author, assistant prof. Nino Sincic (nino.sincic@mef.hr) without undue reservation, to any qualified researcher.

## Supplementary Materials

Supplementary 1 Supplementary data 1: gene expression on the protien level of genes of interest between seminiferous tubules from tumor-free (ST TF) testis and seminiferous tubules with preserved spermatogenesis from TGCT (ST TGCT) samples. To confirm the possibility of fusing these two different tissue groups in a single joined control group, similarity of expression on protien level was tested by statistical analysis of IRS obtained by semi-quantitatibe IHC morphometry performed on each group respectively. Data shown are IRS means ± standard errors. Kolmogorov-Smirnov test was used and no statistical significance was detected.

Supplementary 2 Supplementary data 2: IHC staining information. Depiction of antibodies used to detect relevant proteins of genes of interest, their catalog numbers, manufacturers, dilution used, HIER protocol, secondary antibody information, reaction localization, and information on positive and negative controls.

Supplementary 3 Supplementary data 3: STRING network and functional analysis results. Graph presenting network node, edge, and interaction analysis as well as all significant functional analysis terms (FDA < 0.05).
