# Supplementary figures and images for: In Search of TGCT Biomarkers: A Comprehensive In Silico and Histopathological Analysis
Source: Dis Markers. 2020 Nov 6;2020:8841880. doi: 10.1155/2020/8841880 (PMC7666710; doi:10.1155/2020/8841880)

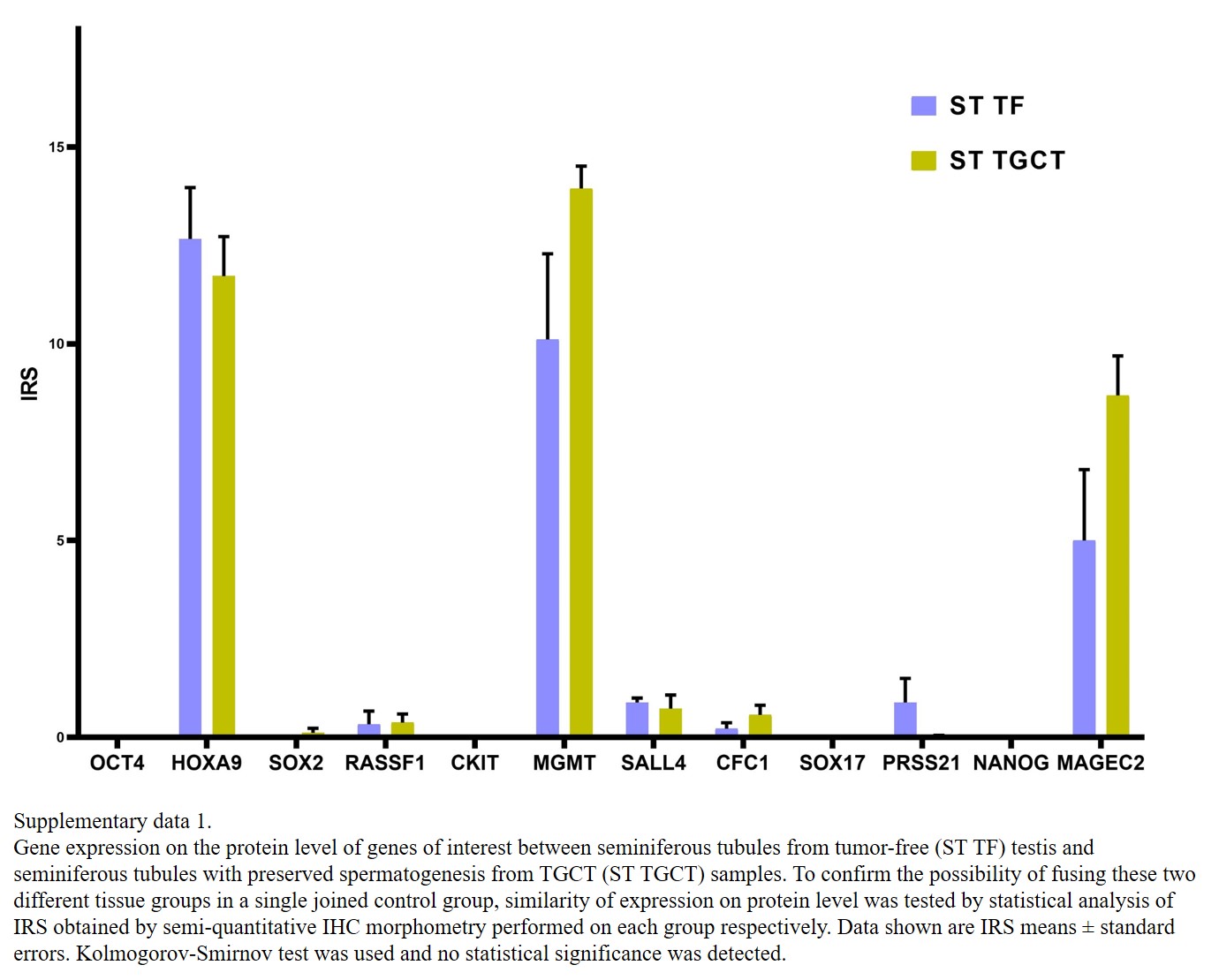

Supplement: Supplementary 1 — Supplementary data 1: gene expression on the protien level of genes of interest between seminiferous tubules from tumor-free (ST TF) testis and seminiferous tubules with preserved spermatogenesis from TGCT (ST TGCT) samples. To confirm the possibility of fusing these two different tissue groups in a single joined control group, similarity of expression on protien level was tested by statistical analysis of IRS obtained by semi-quantitatibe IHC morphometry performed on each group respectively. Data shown are IRS means ± standard errors. Kolmogorov-Smirnov test was used and no statistical significance was detected. [file 8841880.f1.jpg]
