# Supplementary material for: In Search of TGCT Biomarkers: A Comprehensive In Silico and Histopathological Analysis
Source: Dis Markers. 2020 Nov 6;2020:8841880. doi: 10.1155/2020/8841880 (PMC7666710; doi:10.1155/2020/8841880)
Supplement: Supplementary 2 — Supplementary data 2: IHC staining information. Depiction of antibodies used to detect relevant proteins of genes of interest, their catalog numbers, manufacturers, dilution used, HIER protocol, secondary antibody information, reaction localization, and information on positive and negative controls. [file 8841880.f2.docx]

**Table 2.** IHC staining information

Depiction of antibodies used to detect relevant proteins of genes of interest, their catalog numbers, manufacturers, dilution used, HIER protocol, secondary antibody information, reaction localization and information on positive and negative controls.

| Antibody | cat number | Manufacturer | dilution | HIER | Secondary | Reaction localization | Positive control | Negative control |
| --- | --- | --- | --- | --- | --- | --- | --- | --- |
| Anti-RASSF1A | HPA040735 | Atlas Antibodies | 1:200 | pH6 citrate buffer | REAL EnVision Detection System, HRP/DAB, Rabbit/Mouse | cytoplasmic | colon | / |
| Anti-Nanog | AMAb91393 | Atlas Antibodies | 1:1000 | pH9 Tris-EDTA | REAL EnVision Detection System, HRP/DAB, Rabbit/Mouse | nuclear | embryonal carcinoma | pancreas |
| Anti-KITLG | HPA070395 | Atlas Antibodies | 1:350 | pH6 citrate buffer | REAL EnVision Detection System, HRP/DAB, Rabbit/Mouse | cytoplasmic | prostate cancer | pancreas |
| Anti-PRSS21 | HPA008477 | Atlas Antibodies | 1:150 | pH6 citrate buffer | REAL EnVision Detection System, HRP/DAB, Rabbit/Mouse | cytoplasmic | testis | pancreas |
| Anti-SOX2 | AMAb91307 | Atlas Antibodies | 1:1000 | pH9 Tris-EDTA | REAL EnVision Detection System, HRP/DAB, Rabbit/Mouse | nuclear | embryonal carcinoma | pancreas |
| Anti-MGMT | HPA069497 | Atlas Antibodies | 1:750 | pH9 Tris-EDTA | REAL EnVision Detection System, HRP/DAB, Rabbit/Mouse | nuclear | colon | / |
| Anti-HOXA9 | HPA061982 | Atlas Antibodies | 1:1000 | pH9 Tris-EDTA | REAL EnVision Detection System, HRP/DAB, Rabbit/Mouse | nuclear | colon | / |
| Anti-CFC1 | HPA041773 | Atlas Antibodies | 1:1000 | pH6 citrate buffer | REAL EnVision Detection System, HRP/DAB, Rabbit/Mouse | cytoplasmic | pancreas | spleen |
| Anti-SALL4 | HPA015291 | Atlas Antibodies | 1:200 | pH9 Tris-EDTA | REAL EnVision Detection System, HRP/DAB, Rabbit/Mouse | nuclear | testis | pancreas |
| Anti-MAGEC2 | HPA062230 | Atlas Antibodies | 1:750 | pH6 citrate buffer | REAL EnVision Detection System, HRP/DAB, Rabbit/Mouse | nuclear | testis | spleen |
| Anti-SOX17 | ab224637 | Abcam | 1:1000 | pH9 Tris-EDTA | REAL EnVision Detection System, HRP/DAB, Rabbit/Mouse | nuclear | seminoma | skin |
| Anti-Anti-OCT4 | ab109183 | Abcam | 1:1000 | pH6 citrate buffer | REAL EnVision Detection System, HRP/DAB, Rabbit/Mouse | nuclear | seminoma | / |
| Anti-CD117 | A4502 | Dako | 1:100 | En Vision Flex- Target retrival solution, high Ph | REAL EnVision Detection System, HRP/DAB, Rabbit/Mouse | membraneous | seminoma | spleen |
